# A high complex karyotype involving eleven chromosomes including three novel chromosomal aberrations and monoallelic loss of *TP53* in case of follicular lymphoma transformed into B-cell lymphoblastic leukemia

**DOI:** 10.1186/s13039-016-0300-6

**Published:** 2016-12-20

**Authors:** Abdulsamad Wafa, Faten Moassass, Thomas Liehr, Samarth Bhatt, Abdulmunim Aljapawe, Walid Al Achkar

**Affiliations:** 1 Human Genetics Division, Department of Molecular Biology and Biotechnology, Atomic Energy Commission of Syria, P.O. Box 6091, Damascus, Syria; 2 Jena University Hospital, Institute of Human Genetics, Kollegiengasse 10, D-07743 Jena, Germany; 3 Molecular Biology and Biotechnology Department, Mammalians Biology Division, Flow-cytometry Laboratory, Atomic Energy Commission of Syria, P.O. Box 6091 Damascus, Syria

**Keywords:** Follicular lymphoma (FL), t(14;18)(q32;q21), Chromosomal aberration, Clonal evolution, FISH, Prognostic factors

## Abstract

**Background:**

Follicular lymphoma (FL) is one of the most common B-cell non-Hodgkin’s lymphoma (NHL). A subset of FL cases transform into more aggressive malignancies, most often to diffuse large B-cell lymphoma (DLBCL); in addition, lymphoblastic lymphoma and acute lymphoblastic leukemia (ALL) have also been rarely reported. The most common cytogenetic abnormalities associated with FL are translocation t(14;18)(q32;q21) with *BCL2* rearrangements, present in 80–90% of all FL. However, that translocation alone is insufficient to cause FL and additional genomic events specifically leading to this kind of disease are still to be determined. The most frequently reported secondary changes are gains of chromosomes 7p or 7q, Xp, 12q and 18q, as well as losses on 6q and mutations within *BCL2* and/or *BCL6* genes. The presence of additional genomic aberrations, in particular 17p and 6q deletions is more frequent in grade 2 and 3 FL patients and correlated with shorter survival and a higher rate of transformation into DLBCL.

**Case presentation:**

We describe here, an adult FL grade 2 patient that had transformed to B-ALL at diagnosis. Banding cytogenetics, refined by multi-color fluorescence in situ hybridization including array-proven multicolor banding revealed a unique complex karyotype involving eleven chromosomes, translocation t(X;20)(p21.3;q11.2), translocation t(3;20)(q26.2;q12), and a dicentric dic(17;18). Interestingly, the dicentric chromosome led to monosomy of the tumor suppressor gene TP53. The case had an immunophenotype consistent with follicular center cell lymphoma according to the World Health Organization (WHO) recommendations.

**Conclusions:**

To the best of our knowledge, a comparable adult FL grade 2 case that transformed to B-ALL associated with such a complex karyotype and loss of *TP53* was not previously reported. Most of complex aberrations were found simultaneously in approximately 85% of studied malignant cells and the remained cells studied were non-clonal; mechanisms explaining this may be either multiple-step mechanisms or single step in sense of chromothripsis.

**Trial registration:**

Identifying number: 3842. Registered 09 July 2012.

## Background

Follicular lymphoma (FL) is one of the most common B-cell non-Hodgkin’s lymphoma (NHL) with a relatively indolent clinical course, accounting for 20–30% of all NHL cases. The overall survival rate in FL patients is 72–77% for 5 years, with a mean survival of 10 years [1]. A subset of FL cases may transform into more aggressive malignancies; most frequently observed is diffuse large B-cell lymphoma (DLBCL) [2]. Furthermore, lymphoblastic lymphoma and acute lymphoblastic leukemia (ALL) can result rarely from an FL [2–4]. During such processes a more virulent subclonal population of cells emerge, typically associated with the loss of the follicular growth pattern, a rapidly progressive clinical course refractory to treatment, and short survival (commonly of less than 2 years) [5, 6]. Also, this transformation is often associated with the occurrence of secondary chromosomal aberrations [7, 8].

The process of malignant transformation provides an important model for the study of oncogenesis and a number of recurring secondary events are recognized which may be of mechanistic significance [9]. These include acquisition of recurrent chromosomal aberrations like loss of 17p and gain of copy numbers at 12q, inactivation of *CDKN2A* and *CDKN2B*, dysregulation of *c-MYC* and translocations, gains and mutations involving *BCL-6* [9].

The most common cytogenetic abnormalities associated with FL are translocation t(14;18)(q32;q21) with *BCL2* rearrangements, being present in 80–90% of all FL. Other common cytogenetic aberrations are +7, +18, and abnormalities and gene rearrangements in 3q27–28 (*BCL6*), 6q23–26, and 17p [1]. However, the translocation t(14;18)(q32;q21) alone is insufficient to cause FL and those additional genomic events specifically leading to disease are still to be elucidated. Known common cytogenetic aberrations during transformation of FL to DLBCL are translocations and rearrangements of the *BCL2* and *MYC* genes [1].

Here we report a patient with an initial diagnosis of FL grade 2 that transformed to B-ALL. Cytogenetic and molecular cytogenetic analysis revealed a high complex karyotype including three yet unreported chromosomal aberrations, a stable dicentric derivative chromosome and monoallelic loss of the tumor suppressor genes (TSG) *TP53*. The patient was treated with hyper-CVAD but she relapsed many times.

## Case presentation

A 38-year-old female patient without any known personal or familial medical background presented with a 1 month history of fatigue, weakness, loss of weight and fever. Physical examination and CT scan showed mild splenomegaly (data not shown). Initial laboratory evaluation of peripheral blood revealed a white blood cell (WBC) of 3.1 × 10^9^/l (15% were of blasts), red blood cells (RBC) 4.27 × 10^6^/mm^3^, hemoglobin level of 11.7 g/dl and a platelet count of 156 × 10^9^/l. Biochemistry analyses revealed serum lactate dehydrogenase (LDH) value was 893.2 U/l (normal value up to 480 U/l); serum aspartate aminotrasferase (AST) level was 42 U/l (normal up to 45 U/l); and alanine aminotransferase (ALT) level was 122 U/l (normal up to 45 U/l). Total serum protein was within normal range at 7.1 gm/dl (normal value 6.4–8.3 gm/dl) but serum albumin was 4.2 gm/dl (normal value 3.2–5 gm/dl). Ferritin value was 1349 (13–150 ng/ml). Bone marrow aspiration revealed 95% of blasts. At this point the first cytogenetic and immunophenotypic data were determined; simultaneously she had been diagnosed as having B-ALL based on clinical and pathological data. Treatment with hyper-CVAD for overall 10 months was initiated. The patient did not respond to that treatment and suffered from hematuria, right eye vision deterioration, gastrointestinal bleeding, and fever; she received blood transfusion many times, and her peripheral blood (PB) showed pancytopenia. One month after initiation of hyper-CVAD treatment her PB reached a short amelioration of different cell counts (WBC, plt, RBC) together with severe diarrhea, fatigue and weakness. One week later the patient showed pancytopenia again with diarrhea, severe stomach heart burn and fever. After one month the BM smear showed less than 40% blats cells with hypocellularity and BM suppression. She received cortisone, and two months later BM revealed again / still pancytopenia, suppression and myelofibrosis. After another two months BM regenerated and WBC count was 66 × 10^9^/l (90% were blasts). Nonetheless, the patient succumbed due to unknown causes whilst under treatment. Her cousin agreed with scientific evaluation of her case and the study was approved by the ethical committee of the Atomic Energy Commission, Damascus, Syria.

## Results

**Fig. 1:**
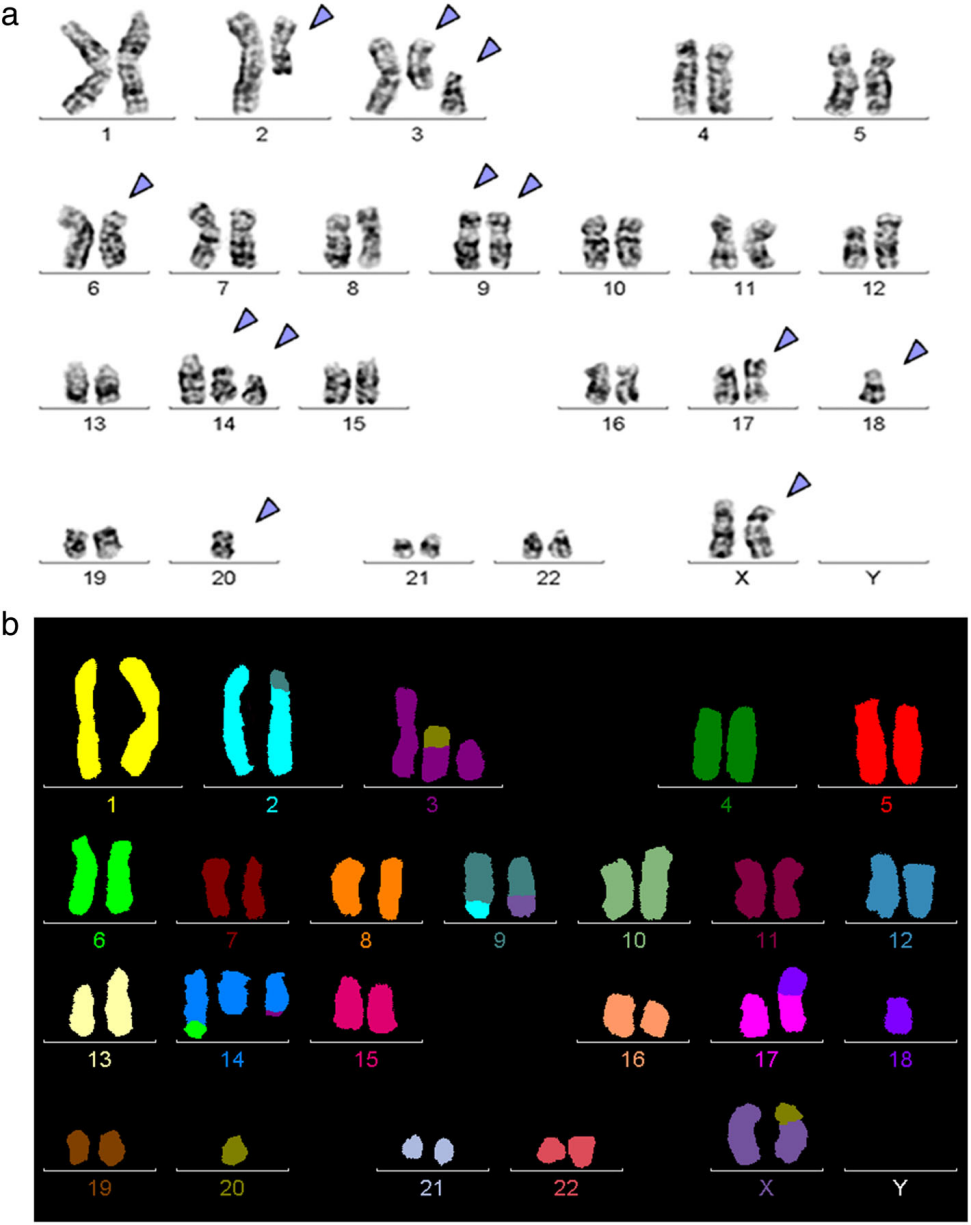
Karyotype and chromosomal aberrations were confirmed using molecular cytogenetic approaches. **a** GTG-banding revealed the following karyotype in 13/20 metaphases: 46,X,der(X)t(X;20)(?;?),t(2;9)(?;?),del(3)(q12),t(6;14)(?;?),der(9)t(X;9)(?;?),der(14)t(6;14)(?;?),del(14)(q?),+der(14)t(3;14)(?;?),+dic(17;18),-17,-18,t(3;20)(?;?). All derivative chromosomes are marked and highlighted by arrow heads. **b** M-FISH confirmed that complexity of the karyotype

**Fig. 2:**
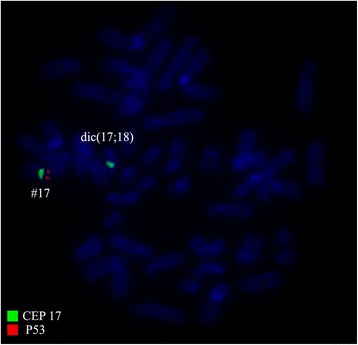
FISH using probes for CEP 17 (*green*) and *TP53* (*red*) showed one green and one red signals on normal chromosome 17; one green signal on der(17) that confirmed the absence of the *TP53* on dic(17;18). Abbreviations: # = chromosome; der = derivative chromosome

**Fig. 3:**
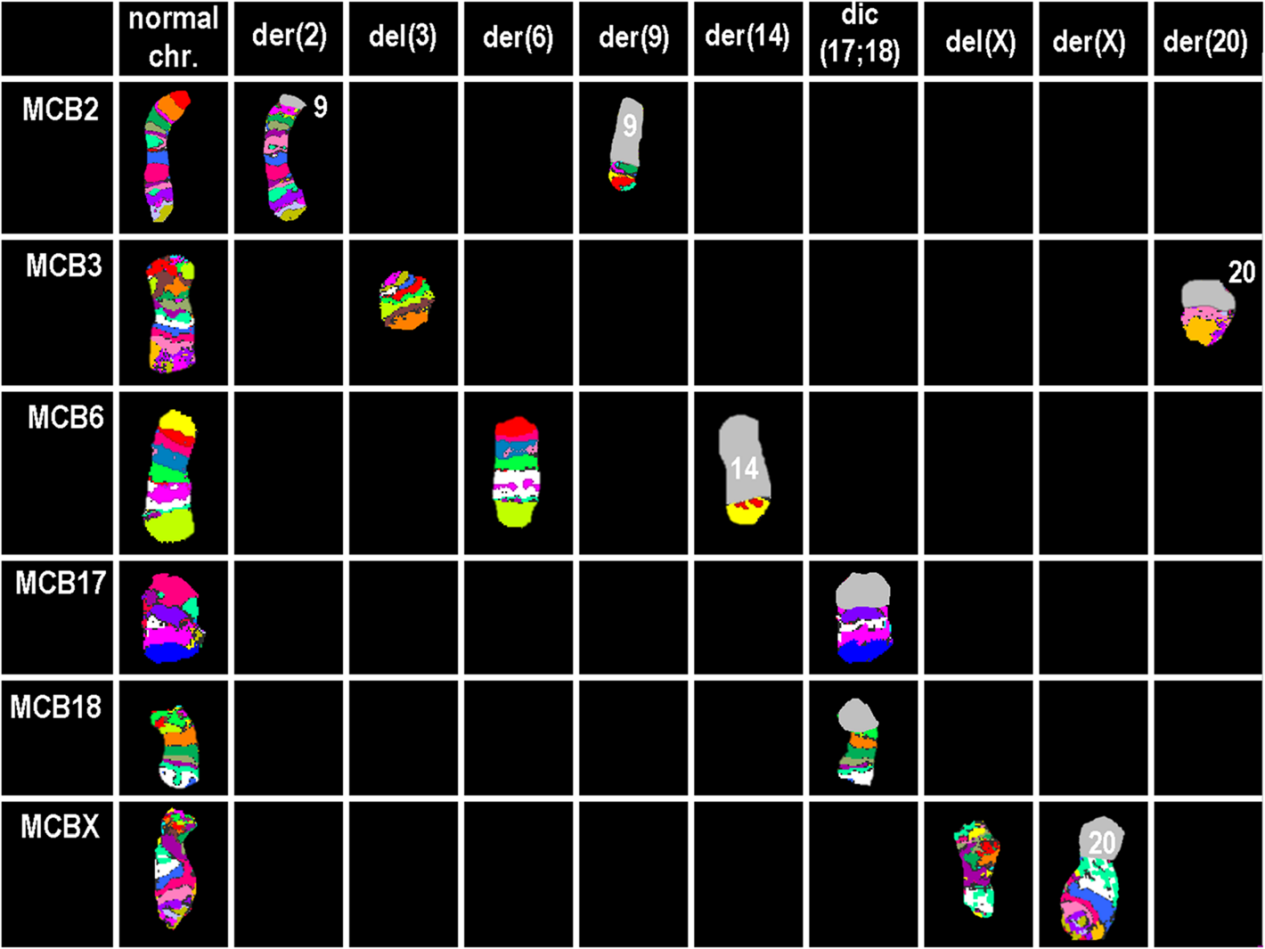
Array-proven multicolor banding (aMCB) results are shown. aMCB results are shown. The normal chromosomes (#) are depicted in the first column on the left side and the derivative of the other chromosomes on the right side of normal chromosomes. The unstained regions when suing chromosome-specific aMCB-probesets on the derivative chromosomes are shown in gray. Abbreviations: # = chromosome; der = derivative chromosome

Banding cytogenetics defined a complex karyotype of 46,X,t(X;20)(?;?),t(2;9)(?;?),del(3)(q?),t(6;14)(?;?),t(X;9)(?;?),t(6;14)(?;?),del(14)(q?),+der(14)t(3;14)(?;?),+dic(17;18),-17,-18,t(3;20)(?;?)[13]/47,X,t(X;20)(?;?),t(2;9)(?;?),del(3)(q?),t(6;14)(?;?),t(X;9)(?;?),t(6;14)(?;?),del(14)(q?),+der(14)t(3;14)(?;?),t(3;20)(?;?)[4]/46,XX[3] (Fig. 1). Further studies were performed based on molecular cytogenetics (Fig. 1). Dual-color-FISH (D-FISH) using specific WCP probes for chromosomes 2, 3, 6, 9, 14, 17, 18, 20 and X (data not shown), m-FISH confirmed a highly complex karyotype (Fig. 1), CEP 17 and 18 probes showed a dicentric chromosome leading to deletions of parts of the short arms of the involved chromosomes (data not shown). The locus-specific probe 17p13 (*TP53*) confirmed the absence of the 17p on the dic(17;18) (Fig. 2). Finally, aMCB using probes for the corresponding chromosomes were performed; Fig. 3). Thus, the following final karyotype was determined:

46,X,t(X;20)(p21.3;q11.2),t(2;9)(p21;q22.3),del(3)(q26.2),t(6;14)(p22.3;q32),t(X;9)(p21.3;q22.3),t(6;14)(p22.3;q32),del(14)(q23),+der(14)(14pter->14q23::3q26.1 ~ 2:),+dic(17;18)(18qter->18p11.21::17p11.2->17qter),-17,-18,t(3;20)(q26.2;q12)[13]/47,X,t(X;20)(p21.3;q11.2),t(2;9)(p21;q22.3),del(3)(q26.2),t(6;14)(p22.3;q32),t(X;9)(p21.3;q22.3),t(6;14)(3p22.3;q32),del(14)(q23),+der(14)(14pter->14q23:: q26.1 ~ 2:),t(3;20)(q26.2;q12)[4]/46,XX[3].

Flow cytometric analysis of peripheral blood specimen characterized this case as B-lineage lymphoproliferative disorder most likely follicular center cell lymphoma according to WHO recommendations. The abnormal cell population (15%) was positive for CD45^dim^, HLA-DR, CD19, CD20, CD10, CD79b, CD123, CD32, CD235a, Lambda and expressed CD7, CD117, CD22, CD23, and CD38 heterogeneously. This cell population was negative for CD34, CD103, CD5, CD11c, sIgD and sIgM.

## Conclusions

We report a cytogenetically highly complex adult FL grade 2 case that transformed to B-ALL with a karyotype involving eleven chromosomes, a dicentric derivative derived from parts of chromosomes 17 and 18 leading to partial monosomy 17p including TSG *TP53* and three yet unreported chromosomal aberrations: t(X;20)(p21.3;q11.2), t(3;20)(q26.2;q12) and dic(17;18)(p11.2;p11.2).

Dicentric chromosomes are normally considered to be instable during mitosis; an idea that was not supported by this and previous own studies [10]. The role of dicentric chromosomes in cancer [11, 12] is still a field to be studied in more detail in future.

FL is regarded as a distinct entity by virtue of its characteristic cellular composition of follicle center cells (centroblasts and centrocytes), uniform immunophenotype (CD10^+^), and common cytogenetic background displaying the translocation t(14;18)(q32;q21) in most of the cases [13]. Since this primary immortalizing event does not render the cells malignant, it is thought that additional secondary aberrations are necessary for tumorigenesis. In FL, the most frequently reported secondary changes are gains of chromosomes 7p or 7q, Xp, 12q and 18q, as well as losses on 6q and possibly mutations of *BCL2* and/or *BCL6* genes. The presence of additional genomic aberrations, in particular 17p and 6q deletions, is more frequent in grade 2 and 3 FL patients and correlated with shorter survival and a higher rate of transformation into DLBCL [14, 15].

The p53 protein, encoded by *TP53* tumor suppressor gene (17p13), lies at the point of convergence of a number of cellular stress pathways [16]. Activation induces cell cycle arrest, DNA repair and, in irreparably damaged cells, apoptosis [9]. Mutations of *TP53* are the most commonly observed in cancer [17]. In FL, mutations are infrequent, while in de novo DLBCL they are more common and may correlate with an adverse prognosis [18]. A recent study suggests that the detection of *TP53* mutations in primary diagnostic specimens of FL without signs of transformation also characterizes a patient subgroup with worse prognosis [19].

Approximately 10–60% of FL cases transform into DLBCL as well as Burkitt’s lymphoma, precursor B lymphoblastic lymphoma and classical type of Hodgkin’s lymphoma [2–4]. However, FL transforming into ALL has been documented in a limited number of cases [3, 4, 20–23]. Some chromosomal aberrations such as gains in chromosomes 2, 3q and 5 have been linked to higher grade transformations and inferior survival [24].

According to the literature, translocation t(2;9) involving short and/or long arms of these chromosomes has been reported in four FL cases to date [25]. Also, translocation t(X;9), deletion del(3)(q26), translocation t(6;14), deletion del(14)(q23), translocation t(3;14) and translocation t(17;18) were previously reported in 3, 3, 3, 1, 54 and 3 FL cases, respectively [25]. Interestingly, translocation t(X;20)(p21.3;q11.2), translocation t(3;20)(q26.2;q12) and dicentric chromosome dic(17;18) has never been described in FL cases. In addition, chromosomal bands such as 2p21, 3q26.2, 6p22.3, 9p22.3, 14q32, 17p11, 18p11, 20q12 and Xp21.3 are involved in chromosomal rearrangements in 14, 16, 5, 19, 1170, 36, 16, 1 and 8 cases, respectively [25]. To the best of our knowledge, a combination of all these rearrangements in one FL case was not previous reported [25]; the present case report is the first one to observe an adult FL transformed to B-ALL with high complexly karyotype.

Al-Tourah et al. [26] established a clinical diagnosis of transformation based on the presence of at least one of the following: sudden rise in LDH, rapid discordant localized nodal growth, new involvement of unusual extranodal sites, new B symptoms can be observed in 30–56% of FL patients and de novo hypercalcemia. However, clinical criteria may not correctly identify all the patients, since some of these symptoms may be associated with FL progression without histological evidence of transformation, while in other cases transformation may not be associated with any of these symptoms [27]. Moreover, FL transformation can also be diagnosed in the absence of all these clinical features at the time of a clinically asymptomatic FL relapse manifested by reappearance of enlarged lymph nodes [27].

The median time from diagnosis to transformation in the reported series ranges from 40 to 66 months, with the earliest transformation reported at 2 months and the latest at 25 years [26, 28, 29]. Transformation may occur at the time of the first or any of the subsequent progressions or recurrences in patients undergoing either expectant follow-up or during and after therapy [27]. Most studies have reported a poor prognosis after transformation with a median duration of survival ranging from 2.5 months to 2 years [27].

Chromothripsis represents one subtype of genomic chaos with highly rearranged chromosomes affecting one or a small number of chromosomes and it was detected in 1% of blood cancers [30]. Genome chaos likely plays a role in the evolution of most cancers [30]. Liu et al. [31] proposed that genome chaos is a common dynamic contributor to cancer progression. However, chromothripsis is not necessarily detectable in all tumors at all stages, it likely occurs during transitional phases (immortalization, transformation, tumor formation, metastasis, and drug resistance) of cancer evolution and is often eliminated or reduced during or after these transitions [32].

In conclusion, we described here a de novo adult FL grade 2 that transformed to B-ALL at diagnosis with a unique complex karyotype involving monosomy TSG *TP53* and three novel uncommon chromosomal aberrations t(X;20)(p21.3;q11.2), t(3;20)(q26.2;q12) and dic(17;18)(p11.2;p11.2). The patient was treated with hyper-CVAD but she relapsed many times. All findings including monosomy of 17p are considered to be an adverse prognostic factor in FL.

## Methods

### Chromosome analysis

Chromosome analysis using GTG-banding according to standard procedures [33] was performed before the treatment started. 20 metaphase cells derived from unstimulated bone marrow culture were analyzed. Karyotype was described according to the International System for Human Cytogenetic Nomenclature (ISCN 2013) [34].

### Molecular cytogenetics

Fluorescence *in situ* hybridization (FISH) using whole chromosome painting (WCP) probes for chromosomes 2, 3, 6, 9, 14, 17, 18, 20 and X (MetaSystems, Altlussheim, Germany), centromere-specific probes (CEP) for chromosomes 17 and 18 (Abbott Molecular/Vysis, USA), and a specific probe for 17p13 (*TP53*) (Q-Biogene, USA) were applied according to manufacturer’s instructions [33]. Multicolor FISH (m-FISH) and FISH using the corresponding chromosome 2, 3, 6, 17, 18 and X specific array-proven multicolor banding (aMCB) probe sets was performed as previously reported [35]. A minimum of 10 metaphase spreads was analyzed, using a fluorescence microscope (AxioImager.Z1 mot, Carl Zeiss Ltd., Hertfordshir, UK) equipped with appropriate filter sets to discriminate between a maximum of five fluorochromes plus the counterstain DAPI (4′,6- diamino-2-phenylindole). Image capture and processing were performed using an ISIS imaging system (MetaSystems).

### Flow cytometric immunophenotype

Immunophenotyping was done using a general panel of fluorescent antibodies against the following antigens typical for different cell lineages and cell types: CD1a, CD2, CD3, CD4, CD5, CD8, CD10, CD11b, CD11c, CD13, CD14, CD15, CD16, CD19, CD20, CD22, CD23, CD32, CD33, CD34, CD38, CD41a, CD45, CD56, CD57, CD64, CD103, CD117, CD123, CD138, CD209, CD235a and CD243; in addition antibodies to Kappa and Lambda light Chains, IgD, sIgM, and HLADr were tested. All antibodies were purchased from BD Biosciences. Samples were analyzed on a BD FACSCalibur™ flow cytometer. Autofluorescence, viability, and isotype controls were included. Flow cytometric data acquisition and analysis were conducted by BD Cellquest™ Pro software.

## Abbreviations

ALL: Acute lymphoblastic leukemia
ALT: Alanine aminotransferase
aMCB: Array-proven multicolor banding
AST: Aspartate aminotransferase
B-ALL: B-acute lymphoblastic leukemia
CEP: Centromeric probe
D-FISH: Dual-color-FISH
DLBCL: Diffuse large B-cell lymphoma
FISH: Fluorescence *in situ* hybridization
FL: Follicular lymphoma
ISCN: International System for Human Cytogenetic Nomenclature
LDH: Lactate dehydrogenase
NHL: Non-Hodgkin’s lymphoma
PB: Peripheral blood
RBC: Red blood cells
TSG: Tumor suppressor genes
WBC: White blood cell
WCP: Probes whole chromosome painting probes
WHO: World Health Organization
